# Dopamine-scrolling: a modern public health challenge requiring urgent attention

**DOI:** 10.1177/17579139251331914

**Published:** 2025-04-12

**Authors:** BT Sharpe, RA Spooner

**Affiliations:** Institute of Psychology, Business, and Human Sciences, University of Chichester, College Lane, Chichester PO19 6PE, UK; Institute of Psychology, Business, and Human Sciences, University of Chichester, Chichester, UK

## Abstract

This manuscript examines the emerging phenomenon of dopamine-scrolling and its implications for public health, particularly regarding mental wellbeing and digital behaviour patterns. While extensive research exists on Internet addiction, problematic social media use, and doom-scrolling, the authors of this paper identify dopamine-scrolling as a distinct behavioural pattern that warrants specific attention from public health professionals and policymakers.

**Figure:**
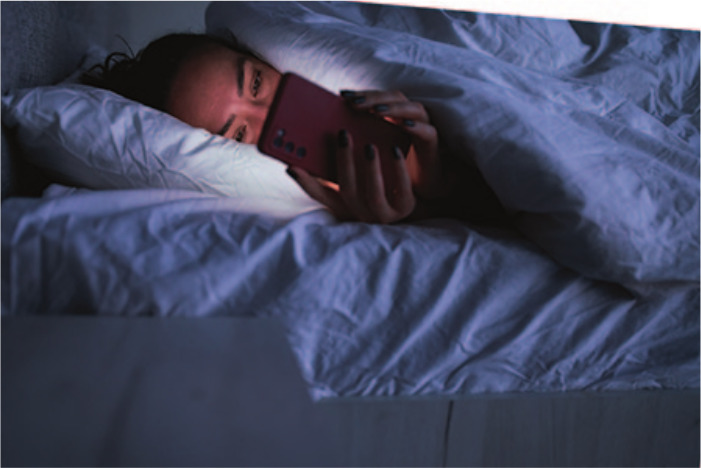


The proliferation of social media platforms has given rise to a distinct behavioural pattern known as dopamine-scrolling – the habitual act of scrolling through social media feeds in pursuit of novel, entertaining content. This behaviour differs fundamentally from other forms of digital engagement, characterised by active seeking of entertaining content, rapid platform switching, and significant time investment.^1^ Recent research indicates that over a billion people spent an average of 3 h scrolling through social media in 2020,^2^ with some countries showing self-reported averages exceeding 4 h daily.^3^

Unlike doom-scrolling, which focuses on negative content,^4^ or Internet Addiction Disorder (IAD), which represents a clinically significant pattern of impairment,^5^ dopamine-scrolling seemingly operates through reward mechanisms and variable reinforcement schedules, making it a unique and potentially habit-forming behaviour.

The behaviour’s prevalence raises significant public health concerns. Studies show that most teenagers report being ‘almost constantly online’,^6^ creating an environment where attention is increasingly fragmented. This extensive usage can lead to various negative outcomes, including mental distraction, degraded social interaction, and potential mental health issues such as anxiety and depression.^7^

Social media platforms employ sophisticated algorithms and design features that capitalise on basic psychological principles to maintain user engagement. These include suggestions, auto-play, pull-to-refresh, infinite scrolling, and social investment mechanisms.^8^ The integration of short-form video content has been particularly effective at triggering psychological patterns that keep users in a continuous scrolling loop.^9^

The neurobiological basis involves small doses of dopamine released with each scrolling motion, coupled with variable reward schedules, which can lead to tolerance development.^10^ This mechanism mirrors the reward uncertainty that makes many behavioural patterns compelling and potentially habit-forming.

Recent research has explored potential solutions. Studies have demonstrated the effectiveness of ethical nudging interventions in minimising time spent on social media and encouraging mindfulness practices.^2^ Practical interventions, such as browser extensions that make social media less compelling and implementing ‘news feed diets’, have shown promise in reducing compulsive scrolling behaviour.^11^

Healthcare providers need to recognise dopamine-scrolling as distinct from other digital behaviours while developing appropriate screening tools and interventions. Educational institutions must develop digital literacy programmes that address this behaviour, helping students understand the mechanisms underlying their social media use while developing strategies for maintaining healthy digital boundaries.^12^

Platform developers and policymakers should consider implementing evidence-based interventions that recognise the particularly vulnerable nature of younger users. Recent policy developments, such as the European Union’s efforts to address digital addiction, represent important steps towards creating protective frameworks.^13^

The way forward requires a multistakeholder approach. This includes the following:

- Development of valid measurement tools for assessing problematic scrolling behaviour.
- Implementation of platform-level features promoting mindful engagement.
- Creation of evidence-based educational interventions.
- Establishment of clear regulatory frameworks protecting vulnerable users.

As we continue navigating an increasingly digital world, understanding and addressing dopamine-scrolling behaviour is becoming crucial for public health. The ongoing evolution of social media platforms suggests this behavioural pattern will likely persist and transform, making it essential to develop effective responses that balance the benefits of digital engagement with the need to protect individual and societal wellbeing.^14^
